# Feeding Habits of European Ground Squirrels in Anthropogenic Habitats in Central Macedonia, Greece

**DOI:** 10.3390/biology14040386

**Published:** 2025-04-08

**Authors:** Stefania Klagkou, Dimitra-Lida Rammou, Ioannis Tsiripidis, Christos Astaras, Dionisios Youlatos

**Affiliations:** 1 Department of Zoology, School of Biology, Aristotle University of Thessaloniki, GR-54124 Thessaloniki, Greece; stefaniaklagkou@gmail.com (S.K.); rdimitra@bio.auth.gr (D.-L.R.); 2 Department of Botany, School of Biology, Aristotle University of Thessaloniki, GR-54124 Thessaloniki, Greece; tsiripid@bio.auth.gr; 3 Forest Research Institute, ELGO-DIMITRA, GR-57006 Vasilika, Greece; christos.astaras@elgo.gr

**Keywords:** *Cynodon dactylon*, dietary flexibility, foraging preferences, ground squirrel conservation, human-modified environment, Mediterranean, *Solanum elaeagnifolium*, *Spermophilus citellus*

## Abstract

The European ground squirrel (*Spermophilus citellus*) is an endangered mammal that inhabits open habitats with low vegetation in central and southeastern Europe. Its southernmost range includes northern Greece, where populations are declining due to habitat degradation and fragmentation. Limited behavioural research on Greek populations underscores the need for studies to support conservation efforts. The present study examined the species’ diet and seasonal changes in food consumption in an anthropogenic area of Central Macedonia, Greece. Between 2022 and 2023, feeding behaviour was documented recording plant parts, taxa, and feeding durations, while plant availability was assessed through surface sampling. The results revealed that *Cynodon*, *Carex*–*Cyperus*, *Salvia*, *Solanum*, and *Plantago* were the dominant plant genera in the species’ diet, while rhizomes and leaves were the most frequently consumed plant parts. Seasonal dietary shifts were observed, with ground squirrels selecting specific plants and plant parts based on availability. Under resource-limited conditions, they adapted by consuming nutritious rhizomes or the less-common, but toxic, *S. elaeagnifolium*. These findings highlight the species’ dietary adaptability to Mediterranean and human-modified environments, providing valuable ecological insights and informing habitat conservation and improvement strategies.

## Simple Summary

The endangered European ground squirrel in northern Greece is threatened by habitat loss and fragmentation. To support conservation efforts, this study investigated their diet and their seasonal change in a human-modified area in Central Macedonia. From 2022 to 2023, we recorded the plants that ground squirrels consumed and found that certain genera, like *Cynodon*, *Carex*–*Cyperus*, *Salvia*, *Solanum*, and *Plantago*, were most common. The squirrels mainly ate rhizomes and leaves, adjusting their diet based on what was available. During periods of food scarcity, they adjusted their diet to include less common or toxic plants. These findings show the species’ ability to adapt its diet in changing environments, and offer useful information for conservation and habitat management.

## 1. Introduction

As anthropogenic habitats rapidly expand globally [1], wildlife in human-modified environments faces numerous challenges, including habitat fragmentation, increased disturbance, and landscape and vegetation structure alterations [2]. These changes significantly affect food availability, which is vital for maintaining physical condition, reproduction, and overall fitness [3,4,5]. In response, wildlife often adapts by incorporating food sources that may not be preferred in natural settings. Foraging decisions in such environments involve trade-offs in timing, location, and diet as animals balance predation risk, energy consumption, and opportunity costs [6,7,8]. This is particularly relevant for hibernators, who must accumulate sufficient energy during a limited active period to survive hibernation, often prioritizing food acquisition over other activities [9,10,11].

The European ground squirrel (*Spermophilus citellus*) is a semi-fossorial and obligate hibernator that has successfully adapted to human-modified habitats [12,13]. The species is endemic to central and southeastern Europe, with its southern range limit extending into northern Greece. There, the species has adapted its activity patterns, foraging, and vigilance behaviours to the arid climatic conditions of the Mediterranean climate [14,15]. In this region, the species faces population decline and habitat loss, resulting in smaller and more-fragmented populations, and is classified as endangered (EN) on the IUCN Red List [16,17,18].

Understanding the dietary habits of small mammals in human-modified environments is crucial for their conservation. Diet influences survival, reproduction, and adaptability, determining how well a species can persist in changing landscapes. In degraded and fragmented habitats, resource availability fluctuates, making dietary flexibility essential for coping with seasonal and environmental challenges. However, no thorough studies on the feeding ecology of European ground squirrels in Greece have been conducted thus far to address these challenges. Most previous studies have been conducted in the natural habitats of northern-ranging populations of the species (in Hungary, Romania, and Serbia), reporting a diverse plant-based diet, consisting primarily of green plant parts, flowers, and seeds [19,20,21,22]. The most representative species are *Achillea* sp., *Festuca* sp., and legumes, with *Achillea millefolium*, various clover species (*Trifolium arvense*, *T. campestre*, *T. medium*, *T. repens*), and dicotyledons, such as *Plantago lanceolata* and *Pimpinella saxifraga*, being particularly important [20,21]. The main plant parts consumed are grass leaves and seeds (Poaceae), dandelion radix (*Taraxacum* sp.), and *A. millefolium* seeds [19,22]. Moreover, insects constitute a significant part of the diet, especially for pregnant females, and small mammals, birds, and reptiles are occasionally consumed [22,23,24]. The species is also known to forage on cultivated crops, cereals, and fallen fruits, further demonstrating its dietary flexibility in human-modified environments [25,26].

As similar data are lacking for the southernmost populations, this study aims to explore the dietary habits of *Spermophilus citellus* at the southernmost edge of its distribution in Central Macedonia, Greece, where human-modified environments under a Mediterranean climate represent the primary habitat of this species [16]. The study seeks to assess selection and seasonal shifts in plant parts and taxa, and to provide insights into the species’ adaptations to human-modified environments in Mediterranean ecosystems. This information will help to elucidate the ecological ways in which the species has adapted to anthropogenic pressures, guiding conservation strategies to mitigate further population declines. Moreover, identifying key food sources may help to inform habitat restoration efforts, ensuring the preservation of critical plant species and improving ecosystem management.

## 2. Materials and Methods

### 2.1. Study Area

**Figure 1:**
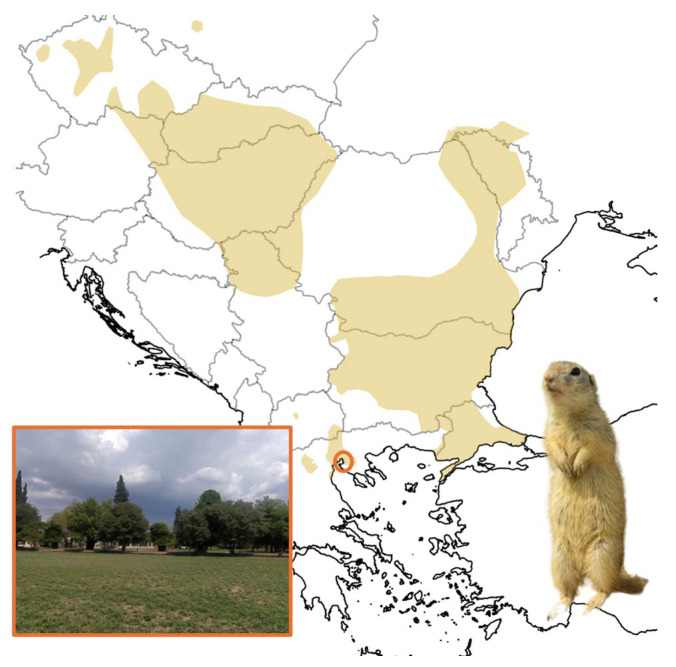
The location of the study area (Thessaloniki, Greece) within the species’ distribution area [18].

Fieldwork was conducted at a European ground squirrel colony present at the School of Agriculture of Aristotle University of Thessaloniki (N 40° 32′, E 22° 59′), located in Thessaloniki, Greece (Figure 1). All observations were carried out within an approximately 0.62 ha fenced area, characterized by low vegetation that is regularly mowed. The site is located near a residential zone with significant anthropogenic influence. Previous studies have reported a relatively high population of European ground squirrels in this colony, with an average abundance of 6.9 ± 6.3 individuals (mean ± SD, late spring census) and an average density of 43.3 ind/ha (late spring census) [15].

### 2.2. Assessment of Plant-Based Food Availability

Vegetation composition and cover were assessed using the quadrat method, with monthly samples in five randomly placed 1 × 1 m (1 m^2^) quadrats. Plant taxa were identified, and cover density was calculated as the mean percentage cover across all quadrats. Identification was performed primarily at the genus level, with species-level identification for distinct taxa, like *Cynodon dactylon* and *Solanum elaeagnifolium*. Differentiating between *Carex* and *Cyperus* was challenging in mowed areas, so both were grouped as *Carex*–*Cyperus*. To explore seasonal diet variation, data were grouped into four periods based on the species’ life cycle: (i) period A (March–April): emergence and reproduction; (ii) period B (May–June): gestation and lactation; (iii) period C (July–August): independence of young; (iv) period D (September–October): preparation for hibernation. For each period, the vegetation composition was calculated as the mean over two months.

### 2.3. Behavioural Data Collection

Our study adhered to the guidelines of the IUCN Position Statement on Research Involving Species at Risk of Extinction [27]. Behavioural data collection covered the entire active period of the species, from March (emergence from hibernation) to October (entry into torpor) [14], but was split between two years: July to October 2022 and March to June 2023. Observations were conducted using binoculars from fixed locations selected to offer maximum visibility across the colony. Only adult individuals, identified by their size and activity, were sampled. As European ground squirrels are diurnal, all observations occurred between 07:30 and 19:30, with slight monthly time adjustments for seasonal daylight variations. Within each month, we collected data during three daily 12 h sessions.

Foraging behaviour was observed using the ad libitum focal sampling method [28]. An arbitrarily selected individual was continuously monitored for 10 min per session, while foraging, with observations terminated either when the 10 min session ended or when the individual was lost from sight (e.g., behind vegetation or inside a burrow). We then shifted to the next available adult individual in sight, paying particular attention to equally sampling all available individuals. During data collection sessions, the food type and the feeding bout duration (in seconds) were recorded. A bout began when food from a specific plant category was acquired, handled, and ingested, and ended when the individual switched to another behaviour (e.g., movement or vigilance) lasting more than 3 s, or changed food type. Based on studies of intermittent locomotion and vigilance in rodents [29], we used the 3 s threshold to define pauses. Data independence was ensured by limiting observations to 10 min per individual, applying the 3 s threshold, and sampling individuals from different areas in the colony [28,29].

During foraging observations, we also recorded data on different food types (plant or animal matter), and the different parts and genera (or species) of the plants consumed (see Table 1).

### 2.4. Statistical Analysis

As food parts differ in the time needed for their handling and consumption, we calibrated for this discrepancy by computing the weighted consumption of each food type and genus. This entailed multiplying the mean consumption duration of each food item by the total number of feeding bouts, and dividing by the total consumption duration across all food types. Non-parametric tests were applied to assess seasonal variation in food consumption and taxonomic composition; the Shapiro–Wilk test indicated deviation from normal distribution [31]. The G test of independence was used to compare food consumption across different food types and plant genera between seasons, followed by a post hoc pairwise G test for seasonal differences [31]. Both analyses were conducted using the RVAideMemoire package v. 0.9-83-7 [32] in R v4.3.2 [33]. Statistical significance was set at *p* < 0.05. Ivlev’s Index was used to evaluate the preference or avoidance of the five most-consumed plant genera, calculated as follows:

E=(r−p)/(r+p)

where *r* represents the proportion of food consumed, and *p* represents the available proportion of food [34]. The index ranges from −1 to +1, with values near +1 indicating strong preference and values near −1 indicating avoidance [34].

## 3. Results

### 3.1. Vegetation Dynamics and Structure

**Figure 2:**
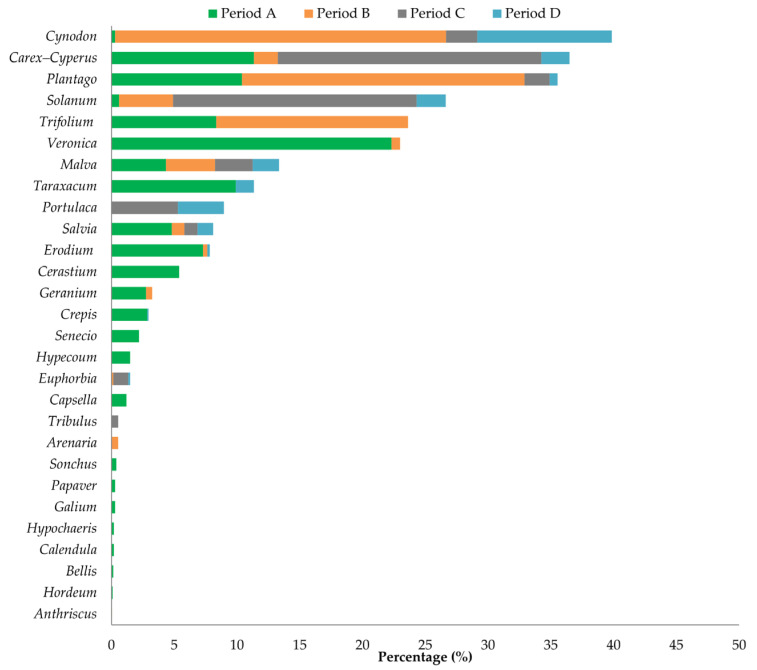
The percentage cover of plant genera recorded in the study area for the different study periods (A: March–April, B: May–June, C: July–August, D: September–October).

Monthly vegetation surveys identified a total of 28 plant genera in the study area (Figure 2). Overall, *Cynodon* (9.91%) and *Carex*–*Cyperus* (9.12%) were the most abundant monocotyledons, and *Solanum* (6.66%) was the most common dicotyledon. Despite high taxonomic diversity, most genera showed low coverage, reflecting the area’s aridity. Seasonal changes revealed that period A had the highest diversity (23 taxa), with relatively low diversity in the periods that followed (period B = 14 taxa, period C = 11 taxa, period D = 14 taxa). Plant taxa coverage changed significantly during the different periods (Figure 2; G = 68.5, *p* < 0.001). Although *Veronica* was the dominant plant in period A (22.35% coverage), it significantly declined during the second period (0.75%) and disappeared altogether as summer progressed. *Plantago* showed considerable coverage in periods A and B (10.45% and 22.50%, respectively), but significantly decreased as summer progressed. *Cynodon* dominated in period B (26.32% coverage), but significantly decreased to 10.73% in period D. *Carex*–*Cyperus* showed variable coverage, with the highest values in periods C and A (21.00% and 11.35%, respectively). *Solanum* was generally under-represented during most periods, but significantly increased to 19.43% coverage in period C.

### 3.2. General Feeding Profile

In terms of food type, the overall food consumption patterns of *S. citellus* revealed the highest intake of rhizomes (38.90%), followed by leaves (28.80%) (Table 2). The latter were consumed twice as often as rhizomes, albeit in threefold shorter feeding bout durations. Stems (12.20%) and seeds (11.10%) followed, while other food types (leaves + stems, inflorescences, and inflorescences + seed + leaves) accounted for less than 5%. Animal matter intake was negligible, being observed only once. Differences in food type consumption were significant (χ^2^ = 111.7, df = 7, *p* < 0.001).

Regarding taxonomic representation, the diet of the species was dominated by a few plant genera, with the weighted consumption percentages and average consumption times detailed in Table 3. *Cynodon* was the most consumed plant (49.40%), consumed more than twice as often as *Carex*–*Cyperus* (21.20%). *Salvia* also represented a considerable percentage of the diet (7.13%), with all other taxa having percentages below 5%. *Cynodon* required the longest bouts to be consumed (mean = 52.5 s), except for animal protein (snails, n = 1–60 s). Differences among the plant taxa consumed were significant (χ^2^ = 650.3, df = 24, *p* < 0.001).

### 3.3. Seasonal Variation in Dietary Patterns

**Figure 3:**
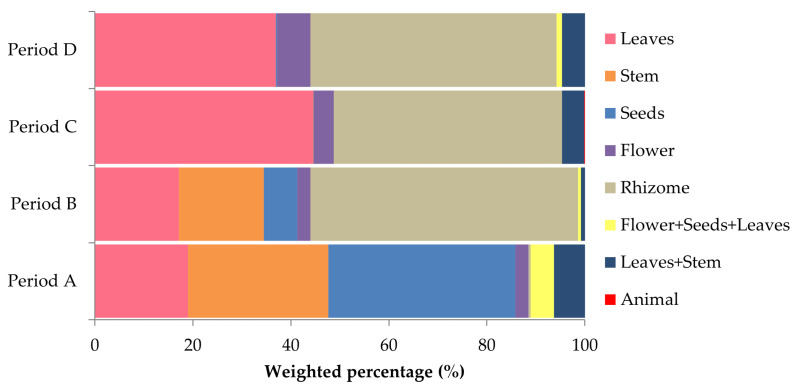
Variation in the consumption of plant food types across periods (A: March–April, B: May–June, C: July–August, D: September–October) by *S. citellus*.

*Spermophilus citellus* exhibited significant seasonal variation in plant food type consumption (G = 328.8, *p* < 0.001; Figure 3). In period A, seeds (38.16%) and stems (28.64%) were mostly consumed. This period was further characterized by minimal rhizome intake (0.48%), which increased significantly during periods B, C, and D (54.66%, 46.55% and 50,20%, respectively) (A vs. B: G = 210.2, *p* < 0.001; A vs. C: G = 160.8, *p* < 0.001; A vs. D: G = 143.1, *p* < 0.001). Leaf consumption was moderate during periods A (18.9%) and B (17.14%), but increased significantly as summer progressed (period C = 44.48%, period D = 36.95%) (A vs. B: G = 114.4, *p* < 0.001; A vs. C: G = 153.3, *p* < 0.001; A vs. D: G = 81.2, *p* < 0.001; B vs. C: G = 180, *p* < 0.001; B vs. D: G = 101.2, *p* < 0.001). After period A, seed consumption progressively declined to 6.86% in period B, and to almost no intake in the subsequent periods (A vs. B: G = 327.8, *p* < 0.001; A vs. C: G = 652.2, *p* < 0.001; A vs. D: G = 326.4, *p* < 0.001). A similar pattern was also observed for stem consumption, as intake decreased to 17.34% in period B, and to almost nothing in periods C and D (A vs. B: G = 380.1, *p* < 0.001; A vs. C: G = 436.8, *p* < 0.001; A vs. D: G = 211.4, *p* < 0.001).

**Figure 4:**
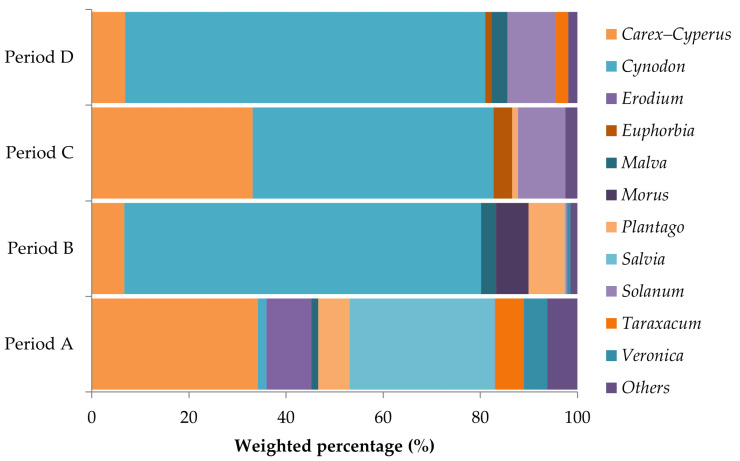
Variation in the consumption of plant genera across periods (A: March–April, B: May–June, C: July–August, D: September–October) by *S. citellus*.

*Spermophilus citellus* exhibited significant seasonal variation in plant taxa consump-tion (G = 348.4, *p* < 0.001; Figure 4). Although *Cynodon* was not consumed just after emergence, it dominated in periods B (73.44%), C (49.85%), and D (74.17%) (A vs. B: G = 131.2, *p* < 0.001; A vs. C: G = 71.4, *p* < 0.001; A vs. D: G = 133.5, *p* < 0.001). Intake of *Carex*–*Cyperus* prevailed in periods A (34.18%) and C (33.14%), but significantly decreased in periods B (6.71%) and D (6.87%) (A vs. B: G = 249.3, *p* < 0.001; A vs. D: G = 245.3, *p* < 0.001; B vs. C: G = 134.6, *p* < 0.001; C vs. D: G = 230.5, *p* < 0.001). *Salvia* represented one of the main food sources of *S. citellus* in period A (29.85%), but its intake significantly decreased in period B (0.26%), and ceased thereafter (A vs. B: G = 439.3, *p* < 0.001). Finally, consumption of *Solanum* was minimal in periods A and B, but moderately increased in periods C (9.72%) and D (9.94%) (A vs. C: G = 128.3, *p* < 0.001; A vs. D: G = 131.5, *p* < 0.001; B vs. C: G = 117.8, *p* < 0.001; B vs. D: G = 120.9, *p* < 0.001).

### 3.4. Preference or Avoidance of Plant Taxa

**Figure 5:**
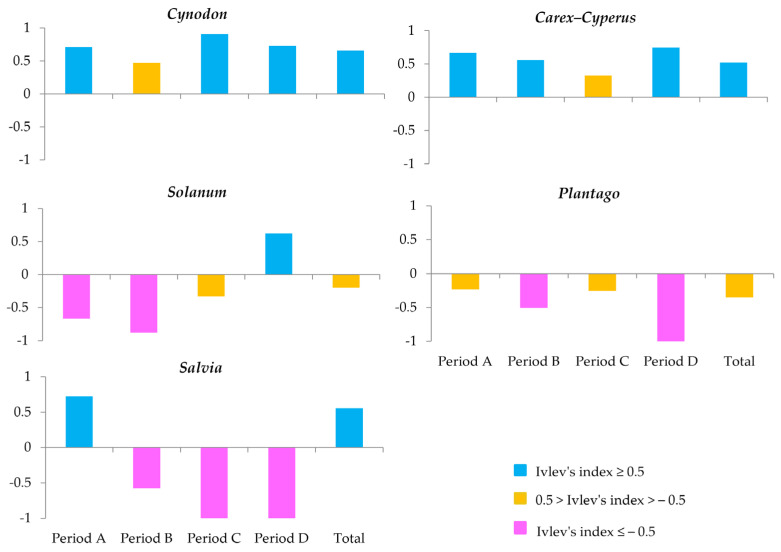
Ivlev’s index for the five most-dominant plant genera in the diet of *S. citellus*, calculated per period and overall. Colour-coding indicates preference (blue), avoidance (purple), or neutrality (yellow).

Ivlev’s Index for the five most-consumed genera by *S. citellus* revealed preferences and avoidances across periods (Figure 5). European ground squirrels showed a strong preference for *Cynodon*, particularly in period C (0.9), with an overall selection index of 0.66. *Carex*–*Cyperus* followed a similar trend, exceeding 0.5 in most periods, but remaining neutral in period C (Figure 5). *Salvia* was well preferred in period A (0.72) but was strongly avoided in periods C and D (−1). Nevertheless, the overall index of 0.55 suggested an overall preference. *Solanum* was avoided in periods A and B, but was preferred in period D, with an overall low avoidance (−0.20). In all periods, *Plantago* seemed to be variably avoided, with a negative overall index (−0.35).

## 4. Discussion

Dietary choices, including food processing and consumption, shape the nutritional specialization, physiology, anatomy, and foraging and vigilance behaviours of animals [2]. Diet analysis also informs key ecological processes, such as habitat selection and species competition. This study examined the dietary habits of the European ground squirrel *Spermophilus citellus* in a human-modified environment in a suburban area of Central Macedonia in Greece, through direct field observations.

In the study area, the vegetation survey recorded 28 plant genera, with taxa related to disturbed, human-modified habitats, such as *Cynodon dactylon*, *Plantago lanceolata*, *Solanum elaeagnifolium*, and *Carex*–*Cyperus*, being the most dominant [35,36,37]. However, as rainfall decreased and temperatures increased during the active period of *Spermophilus citellus*, this diversity significantly diminished, and the relative coverage of some key species, such as *C. dactylon*, decreased [35]. These changes were evident in the transition from *Veronica*, *Carex*–*Cyperus*, *Plantago*, and *Taraxacum* in early spring to *Cynodon* in summer and *Carex*–*Cyperus* and *Solanum* before hibernation in late autumn. *Cynodon* remained dominant due to strong interspecific competition [38], while *Solanum* thrived in dry months, due to its deep-rooted drought resilience [39]. These seasonal shifts indicate a highly disturbed ecosystem influenced by strong climatic shifts, human activities, and the presence of invasive plant species.

Overall, the diet of *Spermophilus citellus* mainly consisted of selected plant parts, with minimal animal intake. Rhizomes, rich in amylum and proteins, represented the primary food source, followed by leaves, stems, and plant reproductive structures. Rhizomes offer essential nutrients and are a drought-resilient food source, especially during periods when surface vegetation dries out [30] or is removed, as in the case of mowing in human-modified habitats, like the one in this study. Although leaves were more frequently consumed, rhizome foraging took nearly three times longer due to tougher tissues and excavation effort, especially in *Cynodon dactylon*, where rhizomes cluster underground. Young leaves, with higher levels of protein and lower levels of secondary metabolites [40], were preferred. Stems provide hydration and nutrients [41] and appear to be essential when energetic demands are high, such as during emergence, reproduction, and pregnancy. Despite its name (*Spermophilus* = seed lover), seeds were not predominant in the diet of the species, except for the period after emergence from hibernation. This intake perhaps underlines the importance of seeds as a source of protein and lipids, although the fleshy parts are usually lower in protein and lipids, but rich in sugars and water [42].

Dietary composition varied seasonally, with the highest plant diversity in the period of early activity and reproduction (period A), due to abundant vegetation. In the period of gestation and lactation (period B), rhizome intake increased as seed availability declined, and higher temperatures led to plant desiccation. During the periods of progressive independence of young individuals (period C) and preparation for hibernation (period D), leaves and rhizomes dominated, as summer drought and continuous anthropogenic impact (mowing) limited plant growth. Comparative studies show that ground squirrels generally prefer leaves, followed by flowers and seeds [43,44], although some species focus more on seeds [45]. *Spermophilus citellus* prefers leaves over seeds and flowers [20], and selectively forages on rhizomes from species of *Poa*, *Cynodon*, and *Plantago*, while previous studies have shown more moderate intake of rhizomes [19]. This study emphasizes the importance of rhizomes in the diet of the species in its southernmost range, indicating an adaptation to the Mediterranean climate and summer drought, as well as to a highly disturbed habitat, where rhizomes are the sole resource during periods of extreme aridity and frequent mowing. *Cynodon dactylon* is highly adapted to frequent trampling and mowing, and these disturbances facilitate the species becoming dominant in semi-natural and human-made environments.

Animal consumption was minimal, with only one recorded instance in period C (July–August), when an individual consumed a snail (Gastropoda). Studies on other ground squirrel species, such as *Xerospermophilus tereticaudus* and *Ictidomys tridecemlineatus*, report higher intake of insects and animal matter in general [43,46]. In Serbia, fecal analysis of *S. citellus* showed that insect and gastropod consumption reached nearly 25% in August–September, though this may be an overestimate, due to the difficulty of digesting chitin [20]. The minimal invertebrate consumption observed in our study site may reflect the rarity of this food type in the diet of this colony, or potential underestimation due to observation limitations. It is always easier to observe the consumption of plant matter rather than the consumption of animal matter, as the latter usually requires different approaches for seizing and handling the prey.

The diet of *Spermophilus citellus* was dominated by grasses and sedges, with *Cynodon* and *Carex*–*Cyperus* exceeding 20% of plant consumption, reflecting their local abundance. Other frequently consumed taxa, such as *Salvia*, *Solanum*, and *Plantago*, also mirrored their availability within the study site, while minimally represented taxa contributed negligibly to the overall diet of the species. This profile suggests opportunistic foraging according to availability to minimize energy expenditure and predation risk. A similar dietary profile, where consumption of the most prevalent plants, including shrubs and invasive annuals, dominates, also characterizes other ground squirrel species, such as *Xerospermophilus mohavensis* and *Urocitellus mollis* in North America [43]. In contrast, the diet of *S. citellus* in northern Greece appears to differ from that recorded in previous studies [19,20,21]. For instance, in northern Serbia, Arok et al. [20] reported lower grass consumption, despite its abundance. These differences may stem from differential regional plant communities, as previous research focused on sites in eastern Europe, whereas the distinct Mediterranean climate and human-modified habitats of suburban areas in northern Greece appear to strongly shape dietary preferences.

*Cynodon dactylon* was the primary plant food for *Spermophilus citellus*, dominating its diet in three of four activity periods (B, C, D). Rhizomes were presumably preferred over leaves and stems due to their high carbohydrate content, supporting energy-demanding activities like burrowing [47]. Despite the high plant diversity in period A (emergence and reproduction), *S. citellus* still sought *C. dactylon*, possibly for its additional nutritional or medicinal properties [48]. During the late summer drought (period D), *C. dactylon* rhizomes remained a vital food source when most vegetation had dried (see also above). While earlier studies noted frequent consumption, they reported a preference for stem buds [19] and little rhizome intake. In some regions, *S. citellus* avoided *C. dactylon*, favouring *Festuca* spp., likely due to grazing competition [21].

*Carex*–*Cyperus* was the second most important food source for *S. citellus*, with peak consumption during periods of high vegetation cover (Periods A and C). Leaves were preferred, likely due to their softness and frequent mowing limiting plant growth. Despite being considered nutritionally inferior to grasses, Cyperaceae provide comparable nutrients and bioactive compounds with antimicrobial and antioxidant properties [49,50,51]. Unlike previous studies reporting rare consumption [19,20], *S. citellus* actively sought these taxa, even at low densities.

*Salvia* was an occasional but important food source for *Spermophilus citellus* during period A (March–April), with the species showing a preference for its energy-rich seeds. The species favoured its seeds, which are rich in phenolic compounds and polyunsaturated fatty acids, providing high energy, especially during post-hibernation and reproduction [52]. Despite being less abundant, this plant species was selectively foraged (Ivlev’s Index > 0.5) post-hibernation and during reproduction [53]. However, it was avoided in later periods (Ivlev’s Index = −1), possibly due to its strong odour from essential oils.

*Solanum elaeagnifolium* is an alien and invasive species in highly disturbed environments, and is toxic to many animals. It represented a moderate proportion of the diet of *Spermophilus citellus*, with increased consumption during the hotter and more arid periods (C, D). In period C, the density of this invasive plant peaked, and in period D, all other plant resources were scarce. *Spermophilus citellus* preferred *S. elaeagnifolium* in pre-hibernation (period D) (Ivlev’s Index > 0.5), likely due to high energy and nutrient demands. During periods A and B, the plant was avoided (Ivlev’s Index < −0.5). The leaves were mostly consumed, with occasional feeding on flowers. Leaves were preferred, presumably due to their lower toxin concentrations, particularly younger leaves, which also possess fewer spines [54]. The increased consumption of flowers may be attributed to their softer tissue, while toxin levels tend to rise in more mature plant structures. Frequent mowing most likely prevented seed production, excluding them from the diet, though *Spermophilus citellus* were observed to consume *Solanum* seeds in nearby areas. Previous studies did not detect consumption of *S. elaeagnifolium*, likely due to the minimal disturbance in the regions studied. The reliance of *S. citellus* on these food sources in human-modified habitats may reflect the adaptability of the species in similar habitats. However, further investigation is required to assess the potential adverse physiological effects associated with toxin ingestion [55].

Some plant genera, like *Plantago*, were also consumed, despite being either avoided or exploited according to availability. This could be related to overall low plant diversity, limiting food choices. *Plantago* leaves are rich in fibre but low in lipids and carbohydrates, making them less significant energy sources [56]. *Spermophilus citellus* primarily consumed *Plantago* during periods A and B, when its density was high, avoiding the need for more energy-demanding foods. Consumption of *Plantago* varied in other areas, depending on environmental conditions [19,20,21].

## 5. Conclusions

In human-modified environments, the survival of *Spermophilus citellus* largely depends on the plant community’s composition and food availability. The dominance of *Cynodon dactylon* and *Carex*–*Cyperus* in the diet of the species is related to their abundance and nutritional content in grassland ecosystems. Ground squirrels in semi-urban environments, like that of the present study, rely heavily on human activities influencing plant communities. The invasive species *Solanum elaeagnifolium*, which competes with other plants, becomes a significant food source during droughts, although the effects of the toxins on the squirrels remain unknown. To support native species and provide more nutritious food, mechanical removal of *S. elaeagnifolium* is recommended. Additionally, reducing mowing during dry months would allow resilient plants to grow, helping to retain morning moisture, and planting more seed-bearing trees could increase food availability. These measures aim to improve the habitat by prioritizing and enhancing key food sources, aligning more closely with the ground squirrel’s natural environment [57]. Ecosystem management that involves maintaining plant diversity and limiting invasive species is essential for the survival and conservation of *S. citellus* in both natural and human-modified ecosystems.

## Figures and Tables

**Table 1:** Variables and definitions of food types and plant parts (based on Crang et al. [30]).

Food Type	Food Part	Definition
Plant matter	Leaves	Lamina, petiole, and sheath
	Stem	Main vegetative axis and lateral branches from which leaves and reproductive organs develop
	Seeds	Includes solitary seeds and those formed on seedheads
	Flower	Includes solitary flowers and inflorescences
	Root	Underground plant organ that functions in anchorage and absorption
	Rhizome	Modified underground stems that grow horizontally at shallow depths beneath the soil surface
	Leaves + Stem	Undistinguishable combination of leaves and stem
	Flower + Seeds + Leaves	Undistinguishable combination of flowers, seeds, and leaves
Animal matter	Animal	Invertebrate or vertebrate body part(s) that could be taxonomically identified by their remains

**Table 2:** Overall mean duration of consumption (sec), total events (N), and weighted percentage of consumption (%) of each food type.

Food Type	Mean Duration (s)	Total Events (N)	Weighted Percentage (%)
Rhizome	73.5	668	38.90
Leaves	23.0	1583	28.80
Stem	29.7	518	12.20
Seeds	35.7	391	11.10
Leaves + Stem	43.7	109	3.77
Flower	17.9	262	3.72
Flower + Seeds + Leaves	21.4	85	1.44
Animal	60.0	1	0.05

**Table 3:** Mean consumption duration (sec), total number of events (N), and weighted consumption (%) per plant genus consumed.

Genus	Mean Duration (s)	Total Events (N)	Weighted Percentage (%)
*Cynodon*	52.5	1154	49.40
*Carex*–*Cyperus*	30.0	867	21.20
*Salvia*	40.1	218	7.13
*Solanum*	20.2	270	4.45
*Plantago*	18.4	285	4.26
*Erodium*	27.3	99	2.20
*Morus*	30.8	86	2.16
*Malva*	27.8	78	1.77
*Taraxacum*	18.2	118	1.75
*Veronica*	19.9	83	1.35
*Euphorbia*	45.7	36	1.34
*Portulaca*	33.2	28	0.75
*Hordeum*	48.7	18	0.71
*Trifolium*	14.5	28	0.33
*Crepis*	17.4	23	0.33
*Geranium*	15.1	20	0.25
*Arenaria*	19.5	14	0.22
*Cerastium*	18.4	10	0.15
*Senecio*	13.7	6	0.06
Gastropoda	60.0	1	0.04
*Tribulus*	40.0	1	0.03
*Capsella*	19.5	2	0.03
*Muscari*	30.0	1	0.02
*Bellis*	9.0	1	<0.01
*Hypecoum*	9.0	1	<0.01

## Data Availability

The raw data supporting the conclusions of this article will be made available by the authors on request.
